# Implementing an intermittent spin-coating strategy to enable bottom-up crystallization in layered halide perovskites

**DOI:** 10.1038/s41467-021-26753-3

**Published:** 2021-11-15

**Authors:** Yajie Yan, Yingguo Yang, Mingli Liang, Mohamed Abdellah, Tõnu Pullerits, Kaibo Zheng, Ziqi Liang

**Affiliations:** 1 Department of Materials Science, Fudan University, Shanghai, 200433 China; 2 Shanghai Synchrotron Radiation Facility (SSRF), Shanghai Advanced Research Institute & Chinese Academy of Sciences, Shanghai, 201204 China; 3 Department of Chemistry, Technical University of Denmark, DK-2800 Kongens Lyngby, Denmark; 4 Department of Chemical Physics and NanoLund, Lund University, Box 124, 22100 Lund, Sweden

**Keywords:** Solar cells, Organic-inorganic nanostructures

## Abstract

Two-dimensional halide perovskites (2D PVSKs) have drawn tremendous attentions owing to their outstanding ambient stability. However, the random orientation of layered crystals severely impedes the out-of-plane carrier transport and limits the solar cell performance. An in-depth understanding coupled with an effective control of the crystallization in 2D PVSKs is the crux for highly efficient and durable devices. In this contribution, we accidentally discovered that the crystallization of 2D PVSKs can be effectively regulated by so-called ′intermittent spin-coating (ISC)′ process. Combined analyses of in(ex)-situ grazing-incidence wide-angle X-ray scattering with time-of-flight secondary ion mass spectrometry distinguish the interface initialized bottom-up crystallization upon ISC treatment from the bi-directional one in the conventional spin-coating process, which results in significantly enhanced crystal orientation and thus facilitated carrier transport as confirmed by both electrical measurements and ultrafast spectroscopies. As a result, the p-i-n architecture planar solar cells based on ISC fabricated paradigm PEA_2_MA_3_Pb_4_I_13_ deliver a respectable efficiency of 11.2% without any treatment, which is three-fold improvement over their spin-coated counterparts and can be further boosted up to 14.0% by NH_4_Cl addition, demonstrating the compatibility of ISC method with other film optimization strategies.

## Abstract (web summary)

Random orientation of layered crystals in 2D perovksites impedes the out-of-plnae carriers transport and hence undermines the photovoltaic perofrmance. Here, the authors discover that the crystallisation can be regulated by intermittent spin-coating strategy.

## Introduction

Two-dimensional lead halide perovskites (2D PVSKs) have emerged as ambient stable photovoltaic materials owing to their unique layered structure. A generic formula of typical 2D Ruddlesden-Popper (RP) PVSKs can be written as L_2_A_*n*−1_Pb_*n*_I_3*n*+1_, wherein the inorganic lattices are sandwiched by organic spacer cations (L^+^) with monovalent cations (A^+^) locating within the cages composed of adjacent eight octahedra^1^. Although the hydrophobic spacer component significantly stabilizes and protects the ionic lattices from degradation upon exposure to moisture, it is achieved at an expense of inefficient charge transfer through thin film, which results from the random crystal alignment and the insulating nature of organic cations^2,3^. Given the comparable in-plane charge transfer capability to 3D PVSKs and the necessity of layer-edge states in exciton dissociation^4^, tremendous efforts have been directed to modulating the crystallization in 2D PVSKs by interfering with their nucleation and growth processes either at interfaces (i.e., solution/substrate and air/solution) or within bulk phases. To be specific, Lewis-base solvent^5^, additives^6,7^, heterogenous atoms^8^, and temperature adjustment^2^ have been exploited to effectively improve the orientation in 2D PVSKs. However, the underlying film-forming mechanism remains largely obscure as a result of more complicated interactions among the organic spacer-containing precursors and the solvent molecules compared to 3D analogues.

**Fig. 1:**
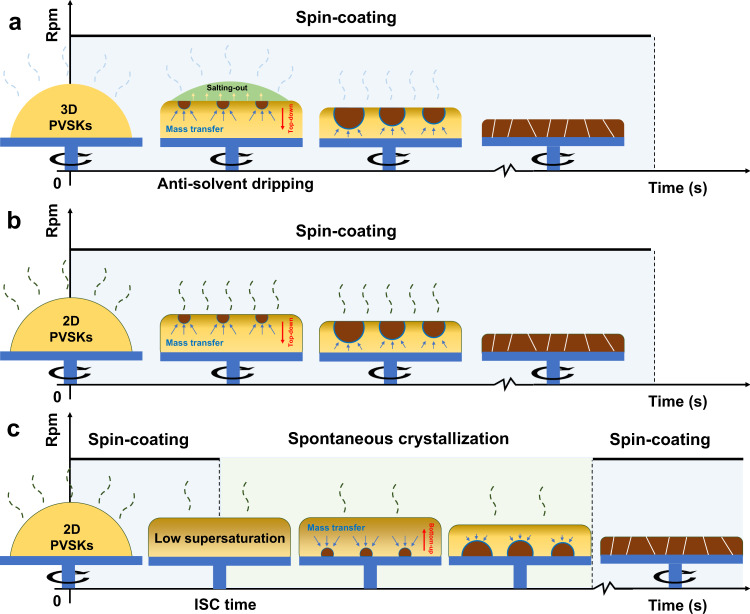
Illustrative of top-down and bottom-up crystallization. Conventional downward crystallization by **a** anti-solvent dripping in 3D PVSKs, **b** direct spin-coating in 2D PVSKs and **c** upward crystallization by ISC method in 2D PVSKs.

In 3D PVSKs, despite a bottom-up crystallization under certain circumstances^9–12^, the top-down nucleation and crystal growth prevail because of the high supersaturation on the surface caused by intense solvent evaporation during spin-coating and anti-solvent salting-out effect as illustrated in Fig. 1a^13–16^. When it comes to 2D PVSKs, the introduced organic cations participate in rather than modify the crystallization, which fundamentally alters the solvent evaporation, the binding process among precursors, and thus the kinetics of film formation. Several works have pioneered and provided important observations in the crystallization of 2D PVSKs^4,17–20^. For example, the Zhou group found in BA_2_MA_3_Pb_4_I_13_ the initial formation of low-*n* (*n* < 4) phases followed by high-*n* ones during spin-coating (SC) via in-situ photoluminescence (PL) spectra, which suggests the favorable kinetics of 2D PVSKs despite their higher formation energy than 3D ones^19^. On the contrary, Kanatzidis, and coworkers recently confirmed by operando grazing-incidence wide-angle X-ray scattering (GIWAXS) technique that the BA_2_MA_2_Pb_3_I_10_ featured a downward crystallization (Fig. 1b) and followed the continuous crystallization model^20^. The divergence between them may lie in either the stoichiometric differences or a variation of preparation methods, which again stresses the complexity even in the same organic cation-based 2D PVSK systems. A universal interpretation is still absent of what dictates the nucleation and crystal growth in 2D PVSKs.

In this work, we accidentally discovered an ′intermittent spin-coating′ (ISC) method that can significantly modulate the crystallization process in layered 2D PVSKs, for instance, a paradigm PEA_2_MA_3_Pb_4_I_13_ (*n* = 4) as shown in Fig. 1c. The precursor solution is first spin-coated for a certain time period—namely, ISC time thereafter, then the wet film is allowed to stand still, and the film-forming is finalized by another spin-coating. Such three-staged film-forming process comprises different roles: (i) the first step homogenizes the precursor solution and sets the initial supersaturation differences between the surface and interface upon entering the second stage, during which (ii) the mass transfer driven by the concentration gradient and the spontaneous solvent evaporation modulate supersaturation rate both on the surface and at the interface, leading to interface initialized nucleation and upward crystal growth; (iii) the last spinning process ensures the fast removal of residual solvent, which prevents the re-dissolution of as-formed crystals and hence avoid the impairment on film morphology. Remarkably, the ISC method enables different optical and structural features along with remarkably enhanced charge carrier transport in the as-prepared films compared with the traditional SC method. The underlying mechanism is unveiled by both in(ex)-situ GIWAXS and time-of-flight secondary ion mass spectrometry (TOF-SIMS) characterizations to establish a bottom-up crystallization model with the optimal ISC treatment, which agrees with the efficient charge transfer from low-*n* (*n* < 4) phases to quasi-3D (*n* > 10) phases as revealed in transient absorption (TA) spectra. These findings collectively contribute to a full comprehension of 2D PVSK crystallization based on the rate of reaching the critical nucleation barrier between air-liquid surface and liquid-substrate interface. As a result, an optimal ISC-8s based device outperforms those SC fabricated counterparts by ~3× enhancement in efficiency. The successful extension of ISC method to other 2D systems further establishes its universality. A complementary inclusion of additive, e.g., NH_4_Cl achieves a remarkable PCE of 14.0%, which demonstrates the compatibility of ISC method with other film optimization strategies.

## Results

### Structural and optical features

In this study, PEA_2_MA_3_Pb_4_I_13_ was prepared from sole DMF solvent without any treatment in order to exclude unexpected effects of solvent and/or additive on the crystallization. It is worth noting that the film thickness remains invariant when the ISC time is not <8 s as confirmed by the step profiler in Supplementary Fig. 1. The film becomes thick, matte and rough as the ISC time decreases, which severely impairs the device performance (Supplementary Fig. 2 and Supplementary Table 1). Therefore, the initial ISC time was set at 8 s to keep the same film thickness for reliable comparison among various ISC times while other ISC times (12, 15, 22, and 30 s) were selected based on experimental observation to ensure both continuous evolution and enough time resolution. All five samples fabricated by various ISC time are denoted as ISC-8s, ISC-12s, ISC-15s, ISC-22s, and ISC-30s (i.e., conventional SC sample), for the sake of brevity.

**Fig. 2:**
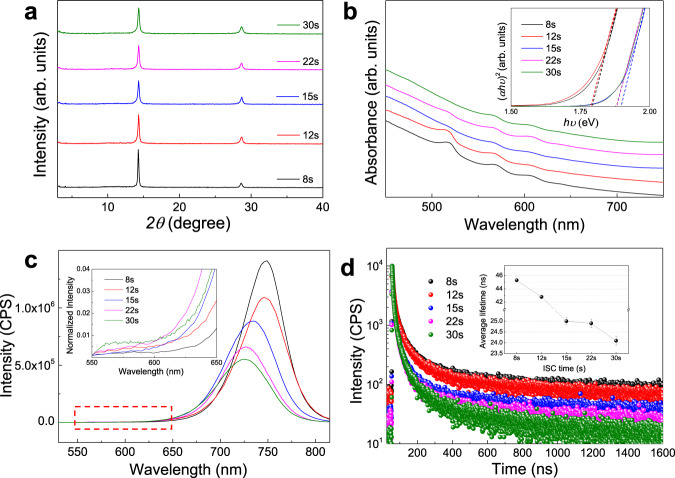
Evolution of crystallinity and phase compositions. PEA_2_MA_3_Pb_4_I_13_ thin films fabricated with various ISC times: **a** XRD patterns and **b** optical absorption spectra with an inset of tauc plots (both are vertically shifted and rescaled for clarity). **c** Steady-state PL spectra with an inset of magnification between 550 and 650 nm, and **d** TRPL kinetics with an inset of derived average lifetimes with standard deviations from three independent films. Thereafter, the black, red, blue, magenta and olive lines (or symbols) represent the ISC-8s, 12 s, 15 s, 22 s and 30 s samples, respectively, unless otherwise indicated.

Figure 2a displays the X-ray diffraction (XRD) patterns of these samples, which share identical peak locations with characteristic peaks around 14.10°, and 28.20° from (111) and (202) facets in 2D PVSKs, indicative of the unobvious influence of the ISC time on the overall crystal structures. On the other hand, the decreasing peak intensity and the narrowing full-width-half-maximum (FWHM) from ISC-8s to ISC-30s samples suggest that the shorter ISC time promotes the crystallization. Thin-film morphology was further evaluated by field-emission scanning electron microscopy (FE-SEM) images as shown in Supplementary Fig. 3. All films exhibit similar and featureless surface topography, which may be attributed to the capping organic layer on surface^4^. The steady-state optical absorption and PL spectra are shown in Fig. 2b and c. The samples display excitonic bands at 530, 580, 610, and 640 nm, corresponding to the *n* value of 1, 2, 3, and 4, respectively, according to our previous reports^5,21,22^. As the ISC time increases from 8 to 30 s, the absorption edge blue-shifts with the optical bandgap (*E*_g_) increasing from ~1.79 to 1.89 eV as derived from the tauc plot (inset of Fig. 2b), which agrees with the main PL peak shift from 760 to 728 nm in Fig. 2c. Such phenomena can be likely interpreted as the existence of larger-*n* phases (i.e., quasi-3D) and/or more efficient carrier transfer from low-*n* to high-*n* phases in short than long ISC samples. Here the quasi-2D and quasi-3D phases refer to those phases with an absorption range from 650 to 700 nm and larger than 700 nm, respectively^21^. Moreover, the monotonously increasing average PL intensity from 5.5 × 10^5^ to 1.4 × 10^6^ counts per second suggests the suppressed non-radiative recombination and thus the enhanced crystallinity in short ISC samples. Interestingly, the optical features in both absorption and PL spectra exhibit a leap between short (≤12 s) and long (>12 s) samples, which will be discussed in the later sections.

Furthermore, the time-resolved PL (TRPL) spectra were acquired to evaluate the influences of ISC method on charge recombination as shown in Fig. 2d. The TRPL kinetics were fitted to a bi-exponential function and the average lifetimes (${\tau }_{{{{{{\rm{ave}}}}}}}$) were estimated to be around 45 and 25 ns for short (≤12 s) and long (>12 s) ISC samples, respectively, which displays a similar plunge as seen above. It should be noted that ${\tau }_{{{{{{\rm{ave}}}}}}}$ only represents the ultimate lifetime when the charge carriers reach the final phases for recombination—in this regard, quasi-3D and quasi-2D for short (≤12 s) and long (>12 s) ISC samples, respectively—due to the measurement limit of time resolution. Therefore, an increasing ${\tau }_{{{{{{\rm{ave}}}}}}}$ corresponds to suppressed trap states in these phases^22^, which accords with the PL results. Based on the above results, the short ISC sample exhibits better crystallinity and possibly transfers charge carriers more efficiently from low-*n* to high-*n* phases than long ISC ones. Still, it remains elusive to explain the sudden and dramatic variations in the optical features and lifetimes, which necessitates an in-depth analysis of crystal orientation and interphase charge transfer dynamics.

### Crystal orientation and *n*-value distribution

**Fig. 3:**
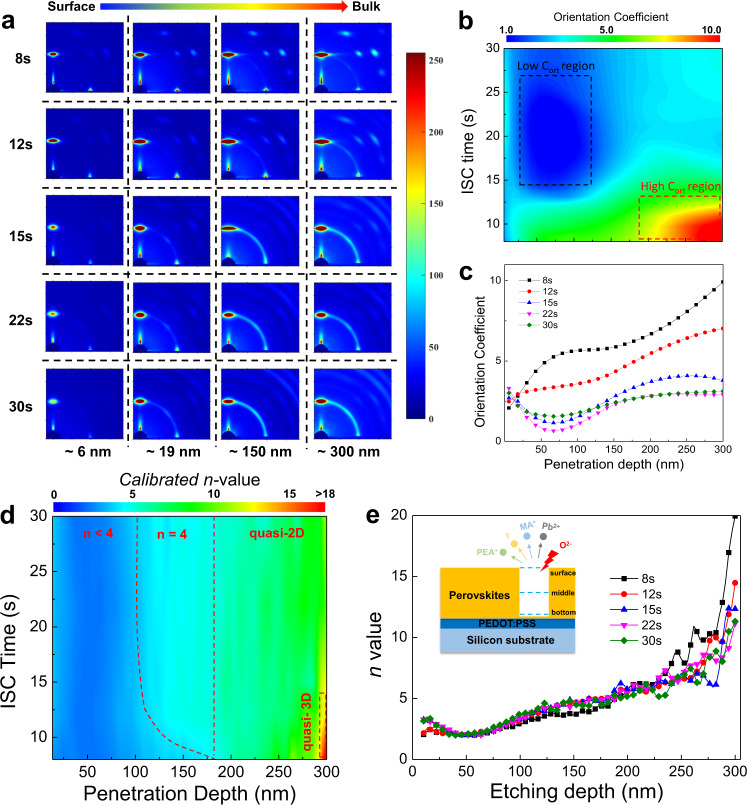
Crystal orientation and phase distribution. **a** Evolution of GIWAXS patterns with various penetration depths and ISC times. **b** Mapping and the corresponding **c** line-cut profiles of *C*_ort_ in PEA_2_MA_3_Pb_4_I_13_ thin films as a function of ISC time and depth. The black(red) dashed box represents the low(high) *C*_ort_ region, respectively. **d** Mapping and **e** the corresponding line-cut profiles of *n* values in PEA_2_MA_3_Pb_4_I_13_ thin films as a function of ISC time and depth. The inset illustrates the ethcing and probing processes on the PEDOT:PSS coated silicon substrates during TOF-SIMS. The red dashed curve and vertical line in **d** separate the whole pane into *n* < 4, *n* = 4 and quasi-2D areas and the red dashed box marks the quasi-3D region.

The GIWAXS technique is a powerful tool for the collection of 2D brag diffractions (spots, arcs, or rings) from certain crystal planes, enabling accurate phase identification and orientation analysis^23–27^. We first adopted ex-situ GIWAXS to investigate the crystallization of PEA-based 2D PVSKs under various ISC times as shown in Fig. 3a. Different incidence angles were applied to vary the penetration depth so as to obtain depth-dependent structural information. Generally, when the incidence angle is less than the critical angle, the X-ray beam can only probe the top surface. As the incidence angle becomes comparable to and even larger than the critical angle, the detection depth surges remarkably. Since the penetration depth does not correlate linearly with the incidence angle, it is difficult to compare the results with the other depth-dependent characterization such as TOF-SIMS, wherein the detection depth is proportional to the etching time. Accordingly, the penetration depth is given as a function of the incidence angle as shown in Supplementary Fig. 4. It was found that the 0.5° is sufficient to penetrate the entire thin films and the incidence angles of 0.1°, 0.18°, 0.3°, and 0.5° were thus used to probe thin films at different depths.

It is observed that an increasing ISC-time leads to attenuating crystal orientation. The ISC-8 and 12 s samples show discrete Bragg scattering spots (or arcs) from {111} and {202} facets, indicative of oriented quasi-2D phases. In contrast, the crystals in ISC-15s, 22 s, and 30 s samples are randomly packed with powder-like diffraction rings. All the samples feature (0k0) peaks at low-**q** region, suggesting the existence of mixed low-*n* phases. In order to quantitatively analyze the texture, the $(\bar{1}1\bar{1})$ and $(\bar{1}11)$ spots around 1 Å^−1^ are selected and integrated along the **q**_**z**_ and **q**_**xy**_ axis with a *χ* range of 5° and the ratio between them (denoted as orientation coefficient, *C*_ort_) are plotted in contour map versus the incidence angle and ISC time as shown in Fig. 3b. The higher the *C*_ort_ is, the more oriented crystals there are in thin films. A high-*C*_ort_ (> 7) region (red dotted box) near the interface is observed in short ISC (≤12 s) samples. Besides, a low-*C*_ort_ (<2) region (blue dotted box) emerges between 0.18° (~20 nm) and 0.3° (~150 nm) when the ISC time is longer than 12 s, indicating much more random orientation in these areas. Moreover, *C*_*ort*_ increases monotonuously from surface to interface for short ISC samples yet exhibits a concave U-shaped trend for long ones as seen from the line cuts in Fig. 3c. Such a phenomenon cannot be fully explained by the GIWAXS analysis alone. Therefore, we resorted to TOF-SIMS to investigate the spatial phase distribution in these samples.

Since the molecular weight of MA fragment falls within the mass range of contaminations such as alkyl and alcohol fragments, the MA signal is severely affected and unable to reflect the corresponding change of content across the films. Therefore, I, Pb and PEA signals having much less overlapped molecular weight with other organic fragments were tracked with the intensity profiles shown in Supplementary Fig. 5. Given the stoichiometry in PEA_2_MA_*n*−1_Pb_*n*_I_3*n*+1_, the intensity ratio of I to PEA (that is, ItP ratio) may be used to reveal the *n*-value variation across the films. Note that we have also calculated the intensity ratio of other components in Supplementary Fig. 6, which exhibits the same trend as ItP ratio. Since the sputtering yield of different components in 2D PVSK films varies a lot and is sensitive to the external factors, the ItP ratio does not show a direct correspondence with *n*-value unless a sample with known *n*-value is simultaneously characterized as the reference. Therefore, we prepared the single crystal of PEA_2_MAPb_2_I_7_ (*n* = 2) and the intensity ratio profiles among I, Pb and PEA are plotted in Supplementary Fig. 7, all of which remain constant across the films. Then, the calibrated *n*-value distributions are plotted in contour map against the etching depth and ISC time as shown in Fig. 3d.

Similar to GIWAXS profiles, the *n* value increases with the etching depth for short ISC samples while it displays a U-shaped distribution for long ISC ones. The ItP ratio (proportional to *n* value) is low in the extremely surficial area, which may be attributed to the thin PEA capping layer as mentioned above^4^. Then, the *n* value increases and descends to its minimum at around 50 nm into the bulk for long ISC samples, after which the ItP ratio begins to increase monotonously. On the other hand, the short ISC samples display lower *n* value adjacent to the surface than long ISC ones. Afterwards, the ItP ratio follows a more flatten trend in the first 50 nm away from the surface and continuously increases across the films, which reaches a higher *n* value at the interface than long ISC samples. The gradient *n*-value distribution in short ISC samples may facilitate exciton splitting and charge transport across the films in comparison to the U-shaped one in long ISC samples. Therefore, two-sided PL emissions were further characterized as shown in Supplementary Fig. 8. For long ISC samples, significant emissions <650 nm emerge as the incident angle of excitation light becomes larger, which indicates the presence of low-*n* phases inside the films. While the short ISC samples feature almost identical PL peak locations from both front and back side regardless of incident angles, suggesting that the photo-excited excitons are more efficiently separated and transferred in short than long ISC samples. Moreover, there is notable redshift (~7 nm) in backside PL peaks from short to long ISC samples, which further verifies the larger *n* values at the interface in the short ISC samples as observed in the TOF-SIMS. The temperature-dependent PL spectra were also conducted and yielded the exciton binding energies (*E*_B_s) of 79.5 ± 6.2 and 133.1 ± 15.0 meV for the ISC-8s and 30 s thin films, respectively, as shown in Supplementary Fig. 9, which are in good agreement with the *E*_B_ for layered PVSKs (for instance, BA_2_MA_3_Pb_4_I_13_) in literature^28^ and confirm the benevolent effect of gradient energy level landscape on exciton separation.

### Mechanistic interpretation

The joint analysis of GIWAXS and TOF-SIMS delivers an interesting picture of orientation and *n*-value distribution. For all ISC samples, the area of orientation evolution overlaps with that of *n*-value distribution. In other words, the higher orientation region corresponds to the larger *n*-value, which is in line with the continuous nucleation model proposed in literature^20^. The cascade *n*-value evolution in short-ISC samples from high-*n* members at the interface to low-*n* ones on the surface with decreasing crystal orientation leads us to derive that the interface initializes nucleation, after which the continuous bottom-up crystallization dominates the crystal growth. On the other hand, the surface still acts as nucleation sites in long ISC-treatments (≥15 s) considering the extremely high supersaturation during long-time spin-coating, which results in bi-directional crystallization into the bulk and the U-shaped *n*-value and orientation distribution as observed. The competition between surface and interface-induced nucleation may be investigated by comparing the critical barrier differences as shown in Eqs. (1) and (2) and Supplementary Fig. 10^29–31^:

1 $${\varDelta G}_{c}^{{{{{{\rm{homo}}}}}}}=\frac{16\pi {\gamma }^{3}{v}^{2}}{3{k}_{B}^{2}{T}^{2}{\left({{{{{\rm{ln}}}}}}S\right)}^{2}}$$

2 $${\varDelta G}_{c}^{{{{{{\rm{heter}}}}}}}={{\varnothing }}\times {\varDelta G}_{c}^{{{{{{\rm{homo}}}}}}}=\frac{\left(2+{\cos }\theta \right){\left(1-{\cos }\theta \right)}^{2}}{4}\times {\varDelta G}_{c}^{{{{{{\rm{homo}}}}}}}$$

where ${\varDelta G}_{c}^{{{{{{\rm{homo}}}}}}}$ and ${\Delta G}_{c}^{{{{{{\rm{heter}}}}}}}$ are the critical barrier for homogenous and heterogeneous nucleation, respectively, *k*_*B*_ is the Boltzmann constant, *T* is the temperature, *γ* is the surface energy, *ν* is the molar volume, *S* is the supersaturation of solution, $\varnothing$ is the correction term and *θ* is the contact angle of the solution on the substrate. Since the surface energy and temperature remain constant, the surface/interface supersaturation ratio and the correction term directly related to the contact angle determine the nucleation priority. The contact angle between PEA based precursor solution and PEDOT:PSS coated substrate was measured to be ca. 11.6° as shown in Supplementary Fig. 11 with the corresponding $\varnothing$ calculated to be 3.1 × 10^−4^, which indicates far less ${\Delta G}_{c}^{{{{{{\rm{heter}}}}}}}$ than ${\Delta G}_{c}^{{{{{{\rm{homo}}}}}}}$. The excellent wettability of precursor solution on PEDOT:PSS substrate renders the heterogenous nucleation at interface much more favorable than that on surface under identical supersaturation condition and thus the dominant upward crystallization as discussed above. We have also investigated the contact angles and GIWAXS patterns on various substrates (e.g., PEDOT:PSS, silicon and PTAA) and cations (such as *n*-BA and octylamine (OA)) based 2D PVSKs as shown in Supplementary Figs. 11–13, all of which evidence the positive correlations between improved wettability and increasing responsiveness to the short ISC method. It is worth noting that the orientation evolution in OA-based system is less sensitive to ISC treatment. This may be attributed to the adverse effect of the elongated alky-chain of OA on the crystallization, which results in insufficient crystallization as verified by the extra (001) signals from the unreacted PbI_2_ (PDF#07-0235) as shown in Supplementary Fig. 12.

**Fig. 4:**
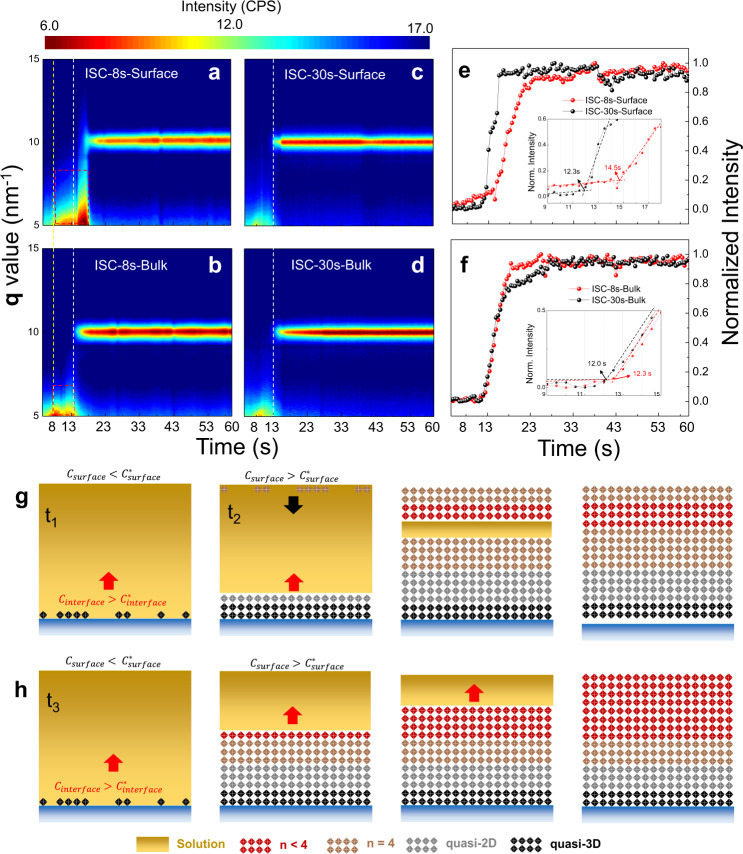
Crystallization comparison of thin-films fabricated with ISC and SC methods. In situ GIWAXS mapping versus time and **q** vector during film formation under **a**, **b** ISC-8s and **c**, **d** SC treatments along with the corresponding line-cuts at ~10 nm^−1^ collected from the **e** surface (0.1°) and **f** bulk area (0.4°). Crystallization illustrations of **g** SC and **h** ISC method. Note *t*_1_ (*t*_3_) and *t*_2_ (*t*_4_) represent the time required to reach the critical concentration for the interface and surface, respectively, in SC (ISC) method. Given the fast crystal growth, the upward crystallization is already completed before the surface reaches *t*_4_ in the ISC method.

In order to directly confirm the interface initialized crystallization, in-situ GIWAXS measurements on both short (8 s) and long (30 s, i.e., SC treatments) ISC samples with different penetration depths were conducted and the false-color map versus **q** and time with the corresponding line cuts were plotted in Fig. 4a–f. In the first spin-coating stage, both samples exhibit a broad scattering intensity around 5 nm^−1^, which originates from the precursor solution^20^. As spin-coating proceeds, the dispersed ring starts to expand as a result of rapid evaporation of the solvent and no noticeable signals at ~10 nm^−1^ is observed (Refer to Supplementary Movies 1−4 for details), which indicates no PVSKs phases generate during this period. When the spin-coating ceases at 8 s, a discontinuous area different from SC samples emerges immediately in the surface areas of ISC-8s sample as highlighted in red dotted box in Fig. 4a. Such metastable intermediate phases do not transform into PVSKs until 14.5 s. On the other hand, the PVSK peak has been identified in the bulk area ~2 s earlier than the surface (Fig. 4b and e), which substantiates the interface initialized crystallization in short ISC samples. When it comes to the SC samples, PVSK phases have already formed around 12 s in both bulk and the surface region as seen in Fig. 4c, d and f, which verifies the bi-directional crystallization in long ISC samples.

Based on these results, a complete picture of crystallization in PEA_2_MA_3_Pb_4_I_13_ is achieved. As schematically illustrated in Fig. 4g and h, during conventional SC process, the concentration in surface area (*C*_*s*_) increases much faster than that at interface (*C*_*i*_) due to the intense evaporation of the solvent. Although the much higher supersaturation on the surface results in low ${\Delta G}_{c}^{{{{{{\rm{homo}}}}}}}$, the extremely low ${\Delta G}_{c}^{{{{{{\rm{heter}}}}}}}$at the interface is also favorable for nucleation, which leads to nearly simultaneous nucleation at both sides as shown in Fig. 4g (i.e., *t*_1_ ≈ *t*_2_). Subsequently, bi-directionally continuous crystallization dominates the crystal growth with gradually decreased *n* value and attenuated orientation into the bulk^20^. With regard to short ISC sample (Fig. 4h), although *C*_*s*_ increases rapidly during the first homogenization stage, the solution is not concentrated enough to trigger homogenous nucleation at the surface. Afterwards, the supersaturation rate on the surface dramatically decreases when the spinning stops and the supersaturation differences between surface and interface gradually vanish as a result of the concentration gradient across the thin wet film, which in turn retards the nucleation on the surface. In this case, the interface reaches the critical supersaturation much earlier than the surface (i.e., *t*_3_ << *t*_4_). Since thin film is left standing with no external perturbance, the overall crystallization is more thermodynamically dictated at this stage, which results in more oriented quasi-3D phases at the interface than SC method. Subsequent monodirectional crystallization yields the observed gradient phase and orientation distribution. If the ISC time continues to descend, the crystallization should be totally different as displayed in Supplementary Fig. 10d. Both the supersaturation on the surface and its differences between surface and interface remain small as a result of insufficient homogenization process and the resulting thick wet film. *C*_*i*_ hardly increases to ${\Delta G}_{c}^{{{{{{\rm{heter}}}}}}}$ because of the limited mass transfer. As a result, *C*_*s*_ gradually increases owing to the spontaneous evaporation and reaches ${\Delta G}_{c}^{{{{{{\rm{homo}}}}}}}\,$(i.e., *t*_5_), which initializes nucleation on the surface and results in downward crystallization.

### Interphase charge transfer dynamics

**Fig. 5:**
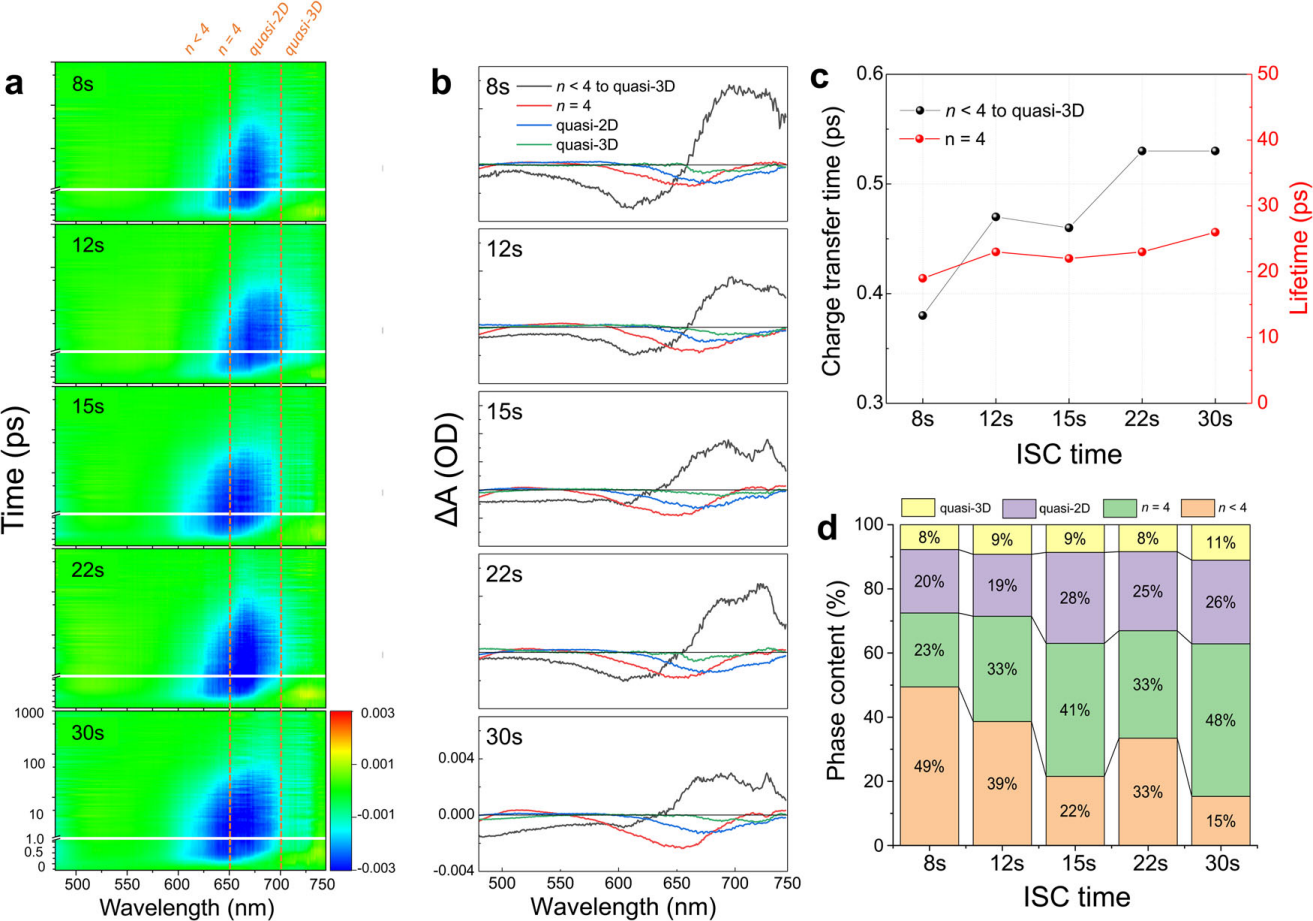
Interphase charge transfer and recombination processes. **a** TA spectrograms of PEA_2_MA_3_Pb_4_I_13_ thin films fabricated with various ISC times. The dashed vertical lines set the absorption borders of *n* < 4, *n* = 4, quasi-2D and quasi-3D phases. **b** SVD fitting profiles symbolizing the charge transfer from *n* < 4 to quasi-3D (black) and the carrier recombination processes in *n* = 4 (red), quasi-2D (blue) and quasi-3D (olive) phases. **c** Derived lifetime in *n* = 4 phase (red dotted line) and carrier transfer time from *n* < 4 to quasi-3D phases (black dotted line) and **d** relative phase contents in percentage upon photo-excitation.

The above results deliver a clear image of the spatial distribution of phases and orientation under various ISC treatments and unveil the origin of the interface initialized upward crystallization in the optimal ISC method. In order to further elucidate the inter-phase charge carrier dynamics in these films, the TA spectra were acquired as shown in Fig. 5. According to the steady optical absorption and PL results, the as-obtained thin films consist of mixed-*n* phases with different *E*_g_s. Therefore, we utilized 400 nm pump pulses with a photon energy larger than the *E*_g_s of all the phases to ensure a non-selective excitation. The distinctive negative ground state bleach (GB) bands at different wavelengths in TA spectrograms correspond to band edge states filling at phases with different *n* values. The GB bands at 610, 640, 650−700, and 750 nm as highlighted in blue areas in Fig. 5a can be assigned to the population of excited charge carriers at *n* = 3, 4, quasi-2D and 3D phases, respectively^21,22^. Besides, there are also trace amounts of GB bands around 530 and 580 nm, which is insignificant yet evidences the excitation of *n* = 1 and 2 phases. After excitation, the photo-generated charge carriers will undergo diffusion and/or inter-phase charge transfer processes^21^. The detailed dynamics are extracted by the singular value decomposition (SVD) fitting of the TA spectra as shown in Fig. 5b. In general, the TA spectra of all samples can be well fitted by four components. The fastest component (black curve) features a negative band at the blue side referring to the recovery of GB at *n* < 4 phases together with a positive band referring to the occurrence of GB at quasi-3D phase. This is a clear fingerprint of ultrafast charge transfer from low-*n* phase to quasi-3D phase after photo-excitation^5^. The other three components show the single negative bands, corresponding to the GB at *n* = 4 (red curve, ~650 nm), quasi-2D (blue curve, 650−700 nm), and quasi-3D phases (olive curve, 700−760 nm), respectively, which reflect the charge carrier recombination within each phase.

We notice that in spite of the relative constant carrier lifetime in high-*n* phases, the inter-phase charge transfer time is shortened with a decrease of ISC time as summarized in Fig. 5c. Efficient charge transfer in shorter-ISC samples can be ascribed to both the ordered structure and the decreased *E*_B_ that facilitate the electronic coupling among various phases, which is consistent with the GIWAXS and TOF-SIMS characterizations. In addition, the efficient inter-phase charge transfer to high-*n* phase also interprets the observed significant variation in the optical features and lifetime between short and long ISC samples as more photo-generated charge carriers will reside at high-*n* phases with prolonged lifetime and recombine in this respect.

In addition to charge transfer, we may also use the absolute intensity ratio of the main GBs among different phases in SVD fittings to estimate their relative content since it represents the initial nonequilibrium distribution of excited charges populating the lowest states of each phase. As shown in Fig. 5d, the contents of *n* < 4 phases in ISC-8s and 12s samples are remarkably higher than those in ISC-15s, 22s and 30s samples. When the ISC time becomes longer, the *n* = 4 phase gradually dominates the bulk films with a shrink in low-*n* phase. Although the low-*n* phase is dominant in short ISC samples, the efficient inter-phase charge transfer as mentioned above guarantees that the final destination of the photo-generated charge carriers should be at quasi-3D phases with higher carrier mobility and lower recombination rate. On the contrary, charge carriers in ISC-15s, 22s, and 30s samples are prone to accumulate within the dominant *n* = 4 and quasi-2D phases due to their suppressed inter-phase charge transfer.

### Electrical characteristics

Besides the interphase charge transport, the space-charge-limited-current (SCLC) method was exploited to evaluate the intrinsic carrier transport of as-prepared thin film^32,33^. It is worth noting that the SCLC measurements are applicable to evaluate the mobility in 2D PVSKs as widely reported^6,34–38^, since the ions are significantly immobilized owing to the organic cations compared with its 3D counterparts^39^. Likewise, the electron-only devices were fabricated to derive the carrier mobility as summarized in Table 1 and Supplementary Fig. 14a, (Refer to Supplementary Table 2 and Supplementary Fig. 15 for fitting details). The mobilities of ISC-8s and ISC-12s films are determined to be ~10^−3^ cm^2^ V^−1^ s^−1^, which are two orders of magnitude higher than those of ISC-15s, 22s and 30s films. Such a striking difference confirms better bulk carrier transfer across the films in short ISC samples. However, it may be too large since the only difference is the ISC time. Thus, the metal–intrinsic semiconductor–metal charge extraction linerar increasing voltage (MIM-CELIV) method was further ultilized to measure the overall carrier mobilities in real devices by considering the influence of interface charge extraction (Supplementary Figs. 14b and 16)^40^.

**Table 1:** Statistic results of SCLC and MIM-CELIV measurements.

ISC time	*n*_trap_ (cm^−3^)	*μ*_SCLC_ (cm^2^ V^−1^ s^−1^)	*μ*_CELIV_ (cm^2^ V^−1^ s^−1^)
8 s	(4.58 ± 0.40) × 10^15^	(1.10 ± 0.26) × 10^−3^	(1.68 ± 0.03) × 10^−4^
12 s	(4.34 ± 0.29) × 10^15^	(2.67 ± 0.20) × 10^−3^	(1.58 ± 0.05) × 10^−4^
15 s	(9.02 ± 1.21) × 10^15^	(1.16 ± 0.14) × 10^−5^	(1.20 ± 0.03) × 10^−4^
22 s	(10.50 ± 1.89) × 10^15^	(0.84 ± 0.16) × 10^−5^	(1.10 ± 0.04) × 10^−4^
30 s	(11.30 ± 0.90) × 10^15^	(0.92 ± 0.05) × 10^−5^	(1.00 ± 0.03) × 10^−4^

The parameters were calculated from three independent devices with the standard deviation from the average; *n*_trap_: trap density derived from the SCLC method, *μ*_SCLC_ and *μ*_CELIV_: mobility determined from the SCLC and CELIV method, respectively.

The carrier mobilities measured by the MIM-CELIV (*μ*_CELIV_) exhibit the same trend as that in SCLC (*μ*_SCLC_). Nevertheless, the *μ*_CELIV_s of all ISC samples fall within a range of 10^−4^ cm^2^ V^−1^ s^−1^ with less obvious differences than that in *μ*_SCLC_s. Moreover, the *μ*_CELIV_s of short ISC samples (8s and 12s) are smaller than corresponding *μ*_SCLC_s while it turns to the opposite trend for long ISC samples. Such a discrepancy lies in which of the bulk or the interface charge transport acts as the bottleneck for overall carrier transportation. As observed in Fig. 3e, the *n* value for the long ISC samples at the ETL side (surface) is higher than those of short ISC ones. Since the conduction band minimum (CBM) of low-*n* members (*n* = 1−4) shifts significantly to deeper energy level as *n* increases^41,42^, higher *n* value on the surface in long ISC samples results in more compatible band alignment with the deep-lying the CBM of PC_61_BM and thus enhanced electron extraction capability compared with short ISC ones. Although the short ISC samples feature highly oriented crystals with the outstanding bulk electron mobility (~10^−3^ cm^2^ V^−1^ s^−1^) as measured by SCLC, the overall mobilities are limited to ~10^−4^ cm^2^ V^−1^ s^−1^ (*μ*_CELIV_) due to its inferior interface charge extraction at ETL side to long ISC samples. On the other hand, despite the significantly suppressed bulk electron transport caused by random orientation, the facilitated electron extraction by PC_61_BM elevates overall mobility from ~10^−5^ cm^2^ V^−1^ s^−1^ to ~10^−4^ cm^2^ V^−1^ s^−1^.

In addition to carrier mobility, the trap densities were also extracted from the SCLC characteristics. The trap densities of ISC-15, 22, and 30 s samples are determined to be (9.02 ± 1.21) × 10^15^, (10.50 ± 1.89) × 10^15^ and (11.30 ± 0.90) × 10^15^ cm^−3^, respectively, all of which are over twice those of ISC-8s ((4.58 ± 0.40) × 10^15^ cm^−3^) and 12 s ((4.34 ± 0.29) × 10^15^ cm^−3^) samples. The above electrical test confirms the enhanced carrier transport in short ISC samples, which is the basis of superior photovoltaic performance to long ISC ones as demonstrated below.

### Solar cell performance

**Fig. 6:**
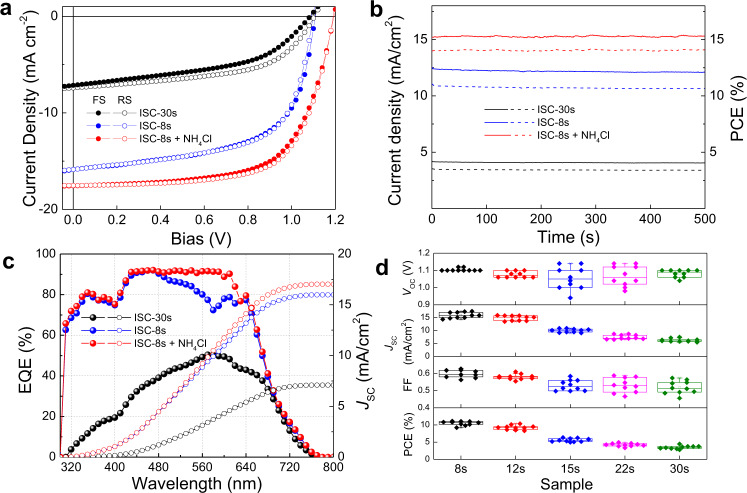
Photovoltaic performance of devices fabricated by ISC versus spin-coating methods. **a** *J−V* characteristics, **b** stabilized outputs and **c** EQE profiles (solid circles) with integrated current density values (empty circles) of ISC-30s (i.e., SC method, black), ISC-8s (blue) and ISC-8s + NH_4_Cl (red) samples. **d** A statistic distribution of device parameters of various ISC times from 10 independent devices. The middle line, upper/lower box limits and upper/lower whiskers in the box plot indicate the median, 25th/75th quartiles, and maximum/minimum, respectively.

The above discussions have unraveled the origins of highly efficient charge transfer upon ISC treatment and as proof-of-concept, the PEA-based 2D RP PSCs were fabricated with a p-i-n architecture of ITO/PEDOT:PSS/PEA_2_MA_3_Pb_4_I_13_/PC_61_BM/BCP/Ag in the absence of any solvent/additive treatment. The representative *J*−*V* characteristics of the optimal ISC-8s and SC (that is, ISC-30s) devices under AM 1.5 G solar simulated light irradiation are shown in Fig. 6a. The optimal ISC device yields a respectable PCE of 11.2% with negligible current hysteresis, which represents 3× improvement over SC fabricated analogues, mainly ascribable to the enhanced orientation and carrier transport as discussed above. The corresponding stabilized outputs were measured by fixing the voltage at the maximum power point as shown in Fig. 6b, which remain steady for 500 s and yield stabilized PCEs of 10.7% and 3.4% for ISC-8s and 30 s device, respectively. To rationalize the accuracy of *J−V* measurements, the integrated *J*_SC_ were calculated from the external quantum efficiency (EQE) spectra in Fig. 6c, which show a small disparity from those obtained in *J-V* characteristics. Besides, the performance distribution of 10 devices fabricated with various ISC time is summarized in Fig. 6d for validation of the reproducibility.

Our previous studies have manifested that the volatile NH_4_Cl additive is conducive to room-temperature crystallization in both 2D and 3D PVSKs yet has insignificant effect on their crystal orientation in comparison to either DMSO or Pb(SCN)_2_^5,22,43^. Therefore, a complementary inclusion of NH_4_Cl was conducted, aiming to further enhance the crystallinity of the ordered PVSK films as obtained from ISC method. Remarkably, the same optimal ISC-device gives rise to an impressive PCE of 14.0% with all simultaneously improved *J*_SC_ of 17.5 mA cm^−2^, *V*_OC_ of 1.2 V and FF of 67% while preserving a negligible current hysteresis factor of 0.03^44^, which demonstrates the compatibility of ISC method with other film optimization strategies. Their corresponding *J–V* characteristics, EQE profiles, and stabilized output are displayed in Fig. 6a−c, respectively. The obtained efficiency is a respectable one among the analogous PEA_2_MA_3_Pb_4_I_13_ (without −F substitution on PEA spacer) based devices as indicated by a careful comparison with literature in Supplementary Table 3.

In order to verify the universality of the ISC method, we have also fabricated photovoltaic devices based on *n*-BA_2_MA_3_Pb_4_I_13_, OA_2_MA_3_Pb_3_I_13_, and F-PEA_2_MA_3_Pb_3_I_13_ with ISC-8s and SC methods and the corresponding performance parameters are summarized in Supplementary Table 4. For both n-BA and F-PEA based devices, though ISC-8s treatments may not be the optimal condition, significant enhancements in the *J*_SC_ by over two-folds prevails for all ISC-8s samples in comparison with SC samples, which accords with the results in Fig. 6d and confirms the applicability of ISC method on other 2D PVSK systems. Unfortunately, the OA-based PVSK thin films displayed poor morphology and the devices yielded no photovoltaic outputs, which possibly results from the unfavorable crystallization as discussed above.

## Discussion

In sum, we have successfully implemented a facile ISC strategy to generate highly oriented 2D PVSK thin films. Compared to the conventional SC process, the ISC method enables a delicate modulation of the supersaturation both on liquid-gas surface and at liquid-substrate interface, which determines the initial nucleation site and the subsequent crystal growth direction. Taking a paradigm PEA_2_MA_3_Pb_4_I_13_ system as an example, we discover that the samples treated by a short ISC time exhibit significantly red-shifted optical features, improved vertical orientation, elevated carrier mobility, and facilitated inter-phase charge transfer, all of which are collectively conducive to enhanced device performance in comparison to reference SC samples. A systematical investigation of the spatial distribution of phases offers an in-depth insight into the crystallization process in 2D PVSKs, which distinguishes the ISC method from conventional SC processes with a dominant interface initialized bottom-up crystallization thanks to the low nucleation barrier at the interface and the retarded supersaturation on the surface. Compared to traditional SC fabricated devices, the optimal neat ISC-8s device delivers a highest PCE of 11.2% with a triple enhancement in stabilized output, and furthermore, an inclusion of NH_4_Cl achieves an outstanding PCE of 14.0%. Further extension to other 2D PVSK systems indicates the universality of ISC method. Overall, this work demonstrates an alternate avenue towards highly oriented 2D PVSK thin films and more importantly sheds light on what dictates the crystallization in 2D PVSKs.

## Methods

### Materials

PbI_2_ (99.9%), CH_3_NH_3_I (99.9%) and CH_3_NH_3_Cl (99.9%) were purchased from Maituowei Ltd. (China). PEA was purchased from Energy Chemical. PbO (99.97%), OA (99%), F-PEA (≥98%) and *n*-BA (≥99.7%) were purchased from Aladdin. PC_61_BM was purchased from Nano-C, Inc. All other chemicals were purchased from J&K Scientific, Ltd. (China). All the reagents were used as received.

### PEA_2_MAPb_2_I_7_ single crystal synthesis

The PEA_2_MAPb_2_I_7_ single crystal was prepared by using cooling precipitation of saturated solution modified from the reported method^45^. CH_3_NH_3_Cl (5 mmol) and PbO powder (10 mmol) were completely dissolved in a mixture of 10 mL HI solution (57 wt% aqueous solution) and 1.7 mL H_3_PO_2_ (50% aqueous solution) under heating and stirring to form a transparent yellow solution **A**. In another beaker, PEA (7 mmol) was neutralized with 5 mL HI solution (57 wt% aqueous solution) in an ice bath, yielding a pale-yellow solution **B**. Solution **B** was added into **A** under continuous stirring and boiling, which finally generated a clear bright yellow solution. Then, the stirring was stopped and the solution was allowed to cool down to room temperature, during which the PEA_2_MAPb_2_I_7_ single crystals precipitated as red rectangular-shaped plates. The crystals were separated by vacuum suction filtration and dried overnight in vacuum oven.

### Thin-film preparation

The pristine PEA_2_MA_3_Pb_4_I_13_ precursor solutions (40 wt%) were prepared by mixing PbI_2_, HI (57 wt% aqueous solution), CH_3_NH_3_I, and PEA at a stoichiometric ratio of 4:2:3:2 in DMF. For NH_4_Cl modification, NH_4_Cl was added into the as-prepared solution in 5 wt%. Then, the precursor solution was stirred under room-temperature at 600 rpm for 1 h. For thin-film fabrication, both the precursor solution w/ and w/o NH_4_Cl treatment were firstly spin-coated at 5000 rpm with an angular acceleration of 5000 rpm/s at room temperature for various ISC times, i.e., 8, 12, 15, 22, 30 s, respectively. Then the spinning process was stopped and the wet film was allowed to stand till its color changed from transparent to light brownish, after which the process was immediately finalized by another spin-coating at 5000 rpm. Note that the standing time for a short ISC sample is strongly affected by the stagnant solvent atmosphere and varies with different samples and ISC timings. As such, the glove box is cleaned by Ar gas replacement for 10 min to reach both H_2_O and O_2_ < 0.1 ppm prior to film deposition. The final process was conducted at 5000 rpm for various ISC times among different samples to keep the total duration of spin-coating identical at 60 s. For short ISC samples, the substrate was firstly spin-coated at 5000 rpm for 8 s (12 s) for ISC-8s (ISC-12s). Then, the spin-coating ceased (requiring about 1 s) and the wet film was left standing for around 4 s (2 s) until the color changed. Finally, the preparation was finished by another spin-coating for 47 s (45 s) at 5000 rpm. For longer ISC samples, since the color has already changed during the first spin-coating stage, the standing time was kept the same as ISC-12s samples at 3 s, after which the film was spin-coated for certain times to keep the total spin-coating time at 60 s. Please refer to Supplementary Movies 5 and 6 for more details. The as-prepared films were stored under inert atmosphere for 1 h to remove residual solvent.

### Film morphology, thickness, and contact angle measurements

FE SEM images coupled with energy-dispersive X-ray elemental analysis were acquired on Philips XL-30 field-emission gun at an accelerating voltage of up to 30 kV. The film thicknesses were measured by Alpha-Step D-500 stylus profiler (KLA-Tenco) and then averaged from 3 measurements. The wettability of PEA_2_MA_3_Pb_4_I_13_ solution on various substrates and the different precursor solution on PEDOT:PSS coated substrate were characterized by a contact angle tester (HARKE-SPCA, HAIKE Test Instrument Company, BEIJING).

### Solar cell fabrication and measurements

ITO substrates were cleaned sequentially in an ultrasonic bath with deionized water, acetone, and isopropyl alcohol (IPA) for 20 min, respectively, and then dried under nitrogen. The substrates were oxidized in UV-ozone for 20 min before use. The PEDOT:PSS layers were spin-coated on the patterned substrates at 3000 rpm for 60 s and annealed at 130 °C for 30 min. The substrates coated PEDOT:PSS were transferred to a N_2_ filled glovebox for making the active layers. After the formation of the perovskite layer, a solution of PC_61_BM (20 mg mL^−1^ in chlorobenzene) was spin-coated at 3000 rpm for 50 s., after which the saturated solution of bathocuproine (BCP) in IPA was spin-coated at 6000 rpm for 30 s. Finally, Ag electrode of 100 nm was thermally deposited with a rate of 0.7 Å s^−1^. The active area as defined shadow mask is ~0.04 cm^2^. The sample was mounted inside a nitrogen-filled sample holder with a quartz optical window for subsequent measurements. The light *J*–*V* curves were measured on a Keithley 2400 source meter unit under AM 1.5 G light illumination with a Newport-Oriel Sol3A Class AAA Solar Simulator (94043 A) operating at an intensity of 100 mW cm^−2^. The light intensity was calibrated by a certified Oriel reference cell (91150 V) and verified with a NREL calibrated, filtered silicon diode (Hamamatsu, S1787-04). The *J–V* profiles were obtained under both forward (−0.2 V → +1.2 V) and reverse (+1.2 V → −0.2 V) scans with a scan rate of 20 mV s^−1^. The EQE spectra were measured on a commercial EQE set-up (QE-R, Enli Technology Co., Ltd). A calibrated silicon diode with a known spectral response was used as a reference.

### Electrical tests

The SCLC measurements were performed on the electron-only devices with a configuration of ITO/SnO_2_/2D PVSKs/PC_61_BM/Ag, in which a bias was scanned from 0 to 3 V with a step of 0.01 V. Based on the SCLC theory, the intrinsic bulk mobility of the insulator is determined by the bias and dielectric constant when the carrier trapping and de-trapping processes are independent of electrical current. The parallel-plate capacitor model can then be adopted to fit this case by Mott–Gurney Law:

3 $$J\times d=\frac{9}{8}\mu {\varepsilon }_{r}{\varepsilon }_{0}\frac{{V}^{2}}{{d}^{2}}=\frac{9}{8}\mu {\varepsilon }_{r}{\varepsilon }_{0}{E}^{2}$$

wherein *ε*_*r*_, *ε*_*0*_, *V* and *d* represent the relative dielectric constant, vacuum permittivity, applied bias and thickness of the active layer, respectively. Prior to entering the space-charge region, the current will suddenly increase owing to the trap filling current when the bias elevates the Fermi level (*E*_*F*_) beyond the defect energy level (*E*_trap_). The trap density (*n*_trap_) can thus be evaluted by trap-fill voltage (*V*_trap_) as shown in Eq. (4):

4 $${n}_{{{{{{\rm{trap}}}}}}}=\frac{2{\varepsilon }_{0}{\varepsilon }_{r}{V}_{{{{{{\rm{trap}}}}}}}}{e{d}^{2}}$$

wherein *e* is the elementary charge and *V*_trap_ is defined as the bias at which the current surges. Therefore, the obtained characteristics were plotted in logarithmic coordinates with the vertical axis representing (*J* × *d*) in A cm^−1^, and the horizontal axis the electric field intensity (*E*) in V cm^−1,^^32,33^. The MIM-CELIV measurements were conducted on the same device structure as working solar cells except that the fresh devices were stored and measured in dark during the whole process. The device sample was first mounted inside a nitrogen-filled sample holder with a quartz optical window. A linearly increasing voltage pulse in reverse bias (the positive probe was connected to the Ag electrode while the negative probe connected to the ITO electrode) was then applied using an arbitrary function generator (Tektronix AFG3021C, 25 MHz bandwidth). The voltage amplitude and ramp were set to be 3 V and 5 × 10^4^ V s^−1^, respectively. The current transients were monitored through a 50 Ω load on a Tektronix oscilloscope (DPO4104B, 1 GHz). The RC constants were observed to be significantly smaller than the time scales of interest. Note that the RC constant values of all devices were estimated to be around 1.7 × 10^−7^ s. This calculation was based on an oscilloscope resistance of 50 Ω and the measured capacitance of the entire diode of ca. 3.4 nF. The *τ*_tr_ was on the order of 10^−6^ s, which was significantly larger than the RC constant, suggesting the reliability of the calculated mobility. The transients were recorded by varying the bias of the voltage pulse and then determined by averaging 64 frames.

### Optical spectrograms

Optical absorption spectra of samples were acquired on Agilent 8453 UV–Visible spectrophotometer. Steady-state photoluminescence was measured using a FluoroMax^@^-4 spectrofluorometer (HORIBA JOBIN YVON, Inc., Edison, NJ) with the excitation beam at 500 nm. The PL intensity was then corrected by absorbed photon numbers at the exciting light wavelength. Every PL peak is an average from four fixed locations in order to rule out the inhomogeneity across thin film. For the two-sided PL spectra, the excitation light was incident from the surface (front) and interface (back) side with different angles (30° and 45°) to enable varied excitation depths into the films. TA experiments were performed by using a femtosecond pump-probe setup in nitrogen atmosphere. Laser pulses (800 nm, 80 fs pulse length, 1 kHz repetition rate) were generated by a regenerative amplifier (Spitfire XP Pro) that was seeded by a femtosecond oscillator (Mai Tai SP, both Spectra Physics). The pump pulses at 400 nm were generated by a BBO crystal as a second harmonic of the laser. The used excitation photon fluxes were 3 × 10^12^ and 1.5 × 10^14^ photons cm^−2^ pulse^−1^. For the probe, we used the super-continuum generation from a thin CaF_2_ plate. The mutual polarization between pump and probe beams was set as the magic angle (54.7°) by placing a Berek compensator in the pump beam. The probe pulse and the reference pulse were dispersed in a spectrograph and detected by a diode array (Pascher Instruments). In order to avoid photo-damage, the sample was moved to a fresh spot after each time delay point. Global SVD analysis was performed with the Glotaran software package (http://glotaran.org). These methods yield more accurate fits of rate constants because they treat the full data set as a whole. A simple sequential decay model with various components is chosen for every fitting. Time-resolved photoluminescence (TRPL) spectra were obtained using a streak camera (Hamamatsu, C6860). The laser source is an amplified titanium/sapphire laser providing 800 nm 35-fs pulses at 2 kHz which is then frequency doubled for 400 nm excitation.

### Crystal structural, orientation and phase composition characterizations

X-ray diffraction pattern data for *2θ* values were collected with a Bruker AX D8 Advance diffractometer with nickel filtered Cu Kα radiation (λ = 1.5406 Å). The ex(in)-situ GIWAXS experiments were carried out at beamline BL14B1 (BL17B1) in Shanghai Synchrotron Radiation Facility (SSRF). Samples were prepared on PEDOT:PSS/PTAA coated glass and ozone treated silicon substrates by using the same preparation conditions as for devices. The data were obtained with an area CCD detector of 3072 by 3072 pixels resolution (225 mm by 225 mm) and a PILATUS detector of 1475 by 1679 pixels resolution (253.7 mm by 288.18 mm). The monochromated energy of the X-ray source was 10 keV. The X-ray wavelength was 1.2378 Å and the incidence angle was set at 0.1°, 0.18°, 0.3°, 0.5° for penetration depth of c.a. 6, 19, 150 and 300 nm, respectively. The 2D GIXRD patterns were analyzed using the FIT 2D software and displayed in scattering vector **q** coordinates. The TOF-SIMS measurements were obtained by TOF.SIMS 5 (IONTOF). The oxygen ion sputtering was used for depth profiling with a focused Bi primary ion pulse for analysis. A area of (240 × 240 µm^2^) is characterized to ensure the uniformity of sampling.

## Supplementary information

Supplementary Information

Description of Additional Supplementary Files

Supplemenatry Movie 1

Supplemenatry Movie 2

Supplemenatry Movie 3

Supplemenatry Movie 4

Supplemenatry Movie 5

Supplemenatry Movie 6

## Data Availability

The data that support the findings of this study are available from the corresponding author upon reasonable request.
