# Supplementary material for: Implementing an intermittent spin-coating strategy to enable bottom-up crystallization in layered halide perovskites
Source: Nat Commun. 2021 Nov 15;12:6603. doi: 10.1038/s41467-021-26753-3 (PMC8593150; doi:10.1038/s41467-021-26753-3)
Supplement: Supplementary file 1 — Supplementary Information [file 41467_2021_26753_MOESM1_ESM.pdf]

Supplemental information

Implementing an intermittent spin-coating strategy to enable bottom-up crystallization in layered halide perovskites

Yajie Yan[1], Yingguo Yang[2], Mingli Liang[3], Mohamed Abdellah[4], Tõnu Pullerits[4], Kaibo Zheng[3,4]*, and Ziqi Liang[1]**

[*][1]Prof. Z. Liang, Y. Yan
Department of Materials Science
Fudan University
Shanghai 200433, China
Email: zqliang@fudan.edu.cn

[*][2]Dr. Yingguo Yang
Shanghai Synchrotron Radiation Facility (SSRF)
Shanghai Advanced Research Institute & Chinese Academy of Sciences
Shanghai 201204, China
Email: yangyingguo@sinap.ac.cn

[*][3]Dr. K. Zheng, M. Liang
Department of Chemistry
Technical University of Denmark
DK-2800 Kongens Lyngby, Denmark
Email: kzheng@kemi.dtu.dk

[4]Dr. M. Abdellah, Dr. K. Zheng, Prof. T. Pullerits
Department of Chemical Physics and NanoLund
Lund University
Box 124, 22100 Lund, Sweden

Results

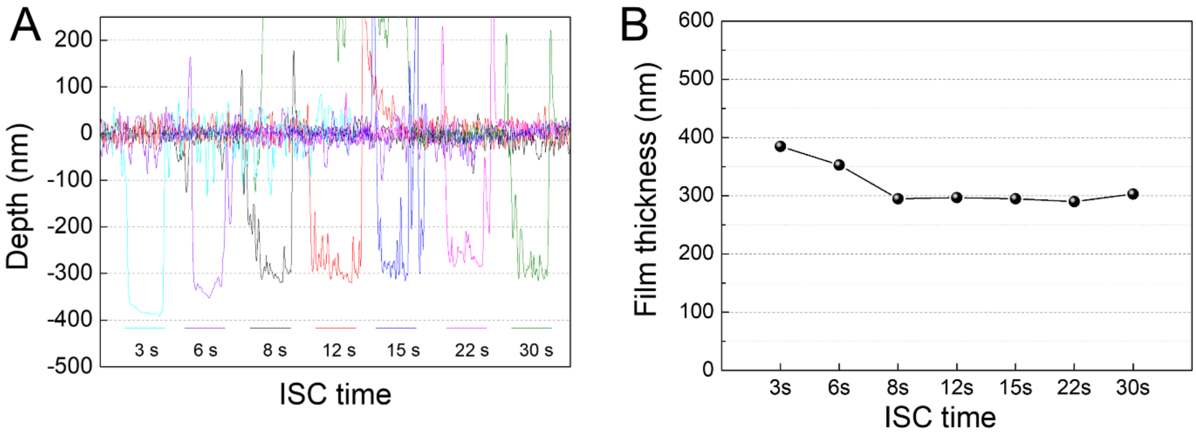

Supplementary Fig. 1 Film thickness determination by step profiler.

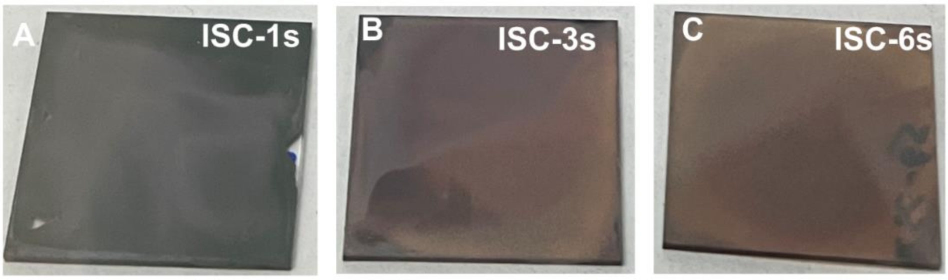

Supplementary Fig. 2 Images of $PEA_2MA_3Pb_4I_{13}$ thin films prepared by (a) ISC-1s, (b) ISC-3s, (c) ISC-6s method, respectively.

Supplementary Table 1 Photovoltaic parameters of devices with different ISC times.

Sample		V_{OC} (V)	J_{SC} (mA/cm^2)	FF	PCE (%)
ISC-6s	RS	0.51 ± 0.03	9.8 ± 1.1	0.41 ± 0.02	2.02 ± 0.20
	FS	0.45 ± 0.03	10.0 ± 0.5	0.40 ± 0.02	1.84 ± 0.21
ISC-3s	RS	0.57 ± 0.05	1.92 ± 0.76	0.30 ± 0.06	0.33 ± 0.08
	FS	0.41 ± 0.11	1.42 ± 0.88	0.22 ± 0.03	0.14 ± 0.12
ISC-1s	RS				
	FS				

Note: a) Every parameter was averaged from 3 solar cells to provide standard deviations from the average. b) All devices fabricated with ISC-1s method die without photovoltaic output.

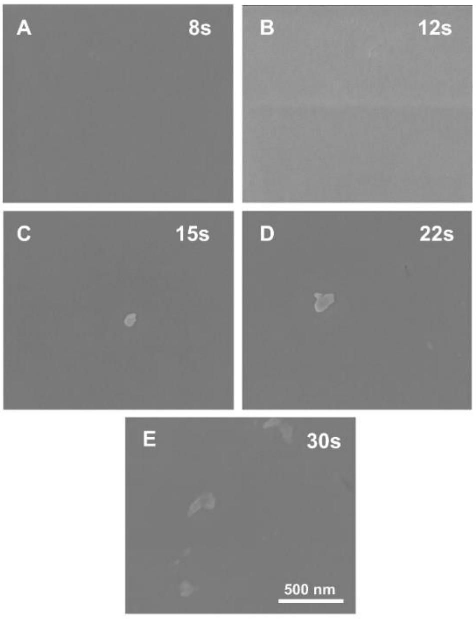

Supplementary Fig. 3 Film topography variations by FE-SEM imaging.

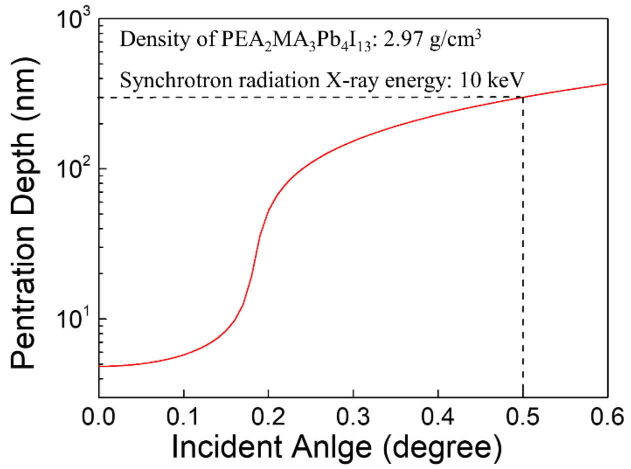

Supplementary Fig. 4 Correlation between X-ray penetration depth and incident angle.

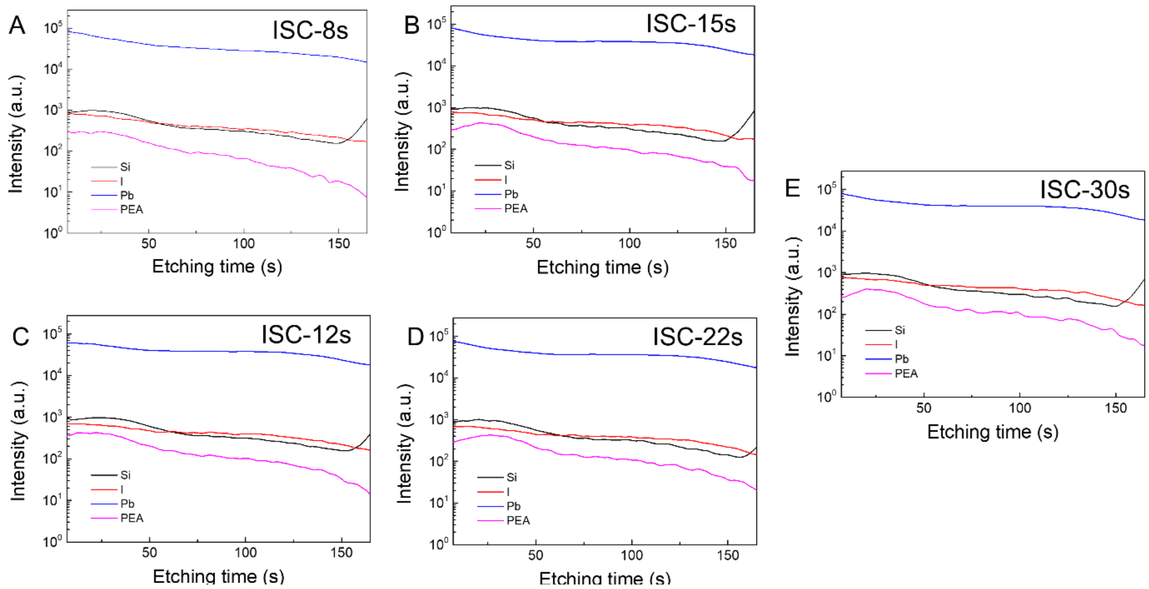

Supplementary Fig. 5 Raw intensity profiles of Si, I, Pb and PEA of in a) ISC-8s, b) 12s, c) 15s, d) 22s, e) 30s samples measured by TOF-SIMS.

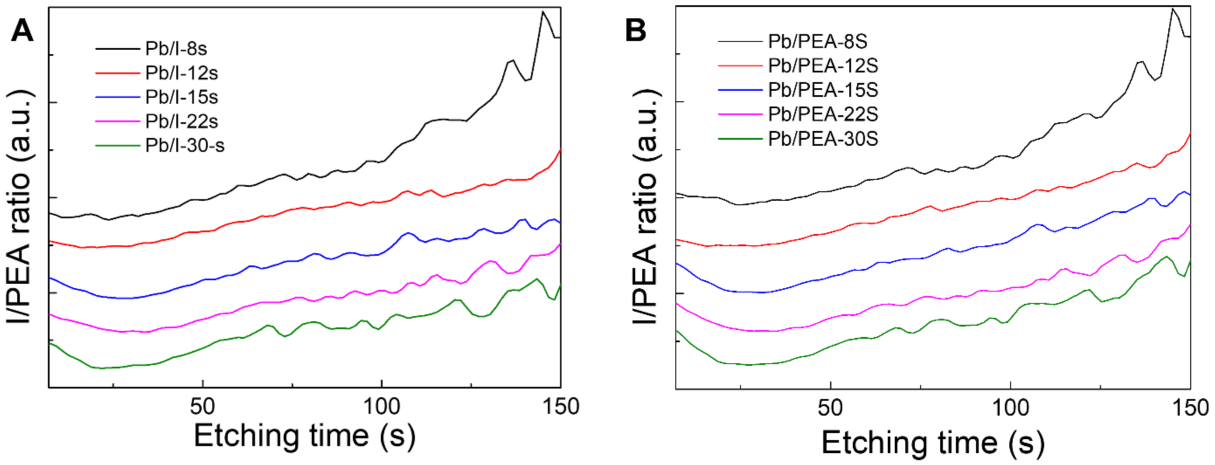

Supplementary Fig. 6 Intensity ratio of (a) I/PEA and (b) Pb/PEA with different etching times.

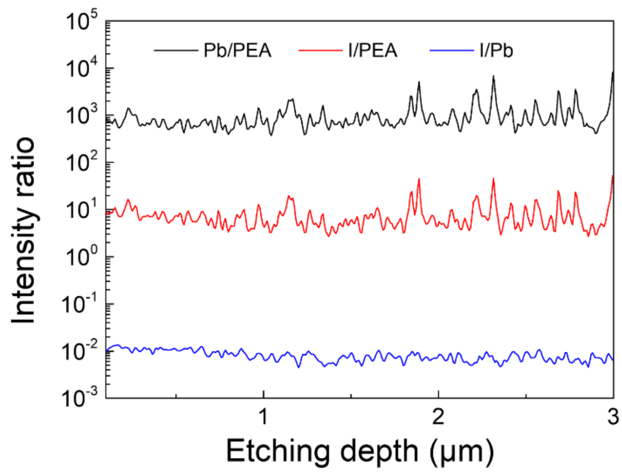

Supplementary Fig. 7 Depth-dependent intensity ratio between different compositions in $PEA_2MAPb_2I_7$ single crystal.

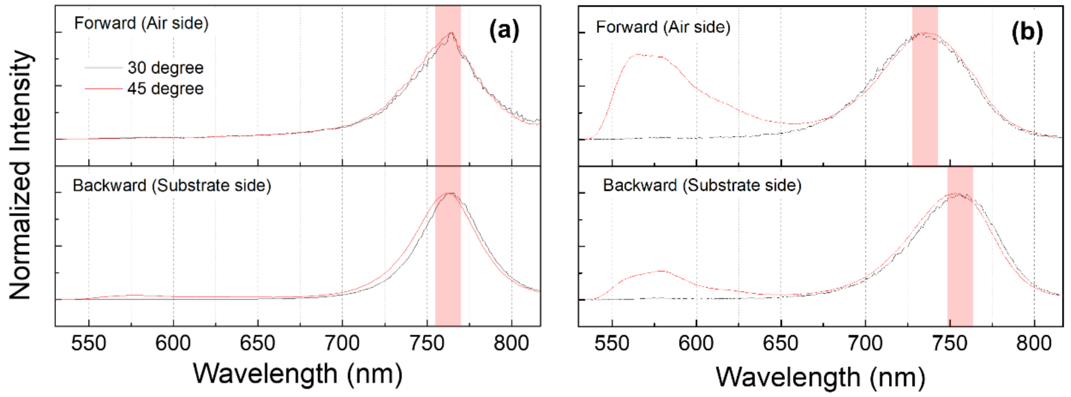

Supplementary Fig. 8 Forward and backward PL emissions of (A) ISC-8s and (B) ISC-30s thin films with incident angles of 15° (black line) and 45° (red line), respectively.

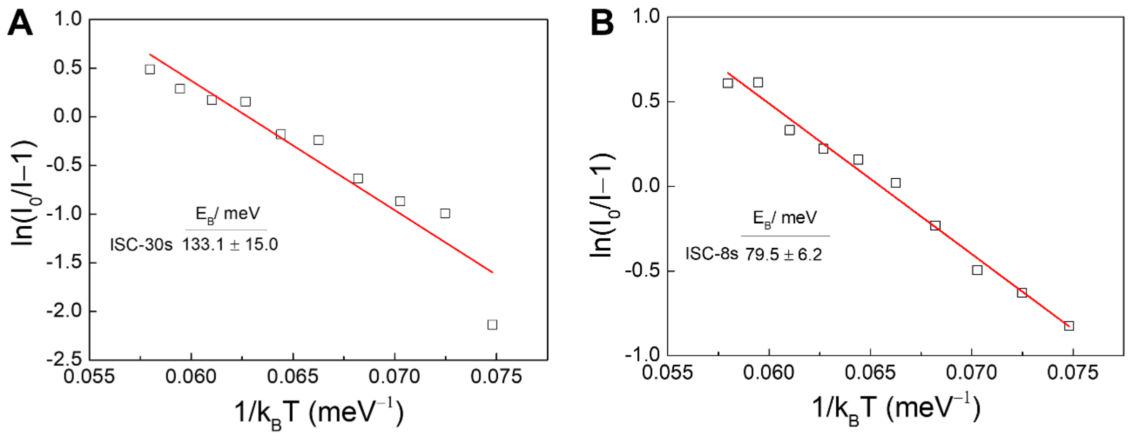

Supplementary Fig. 9 Binding energy determination of (a) ISC-30s and (b) 8s samples with standard errors.

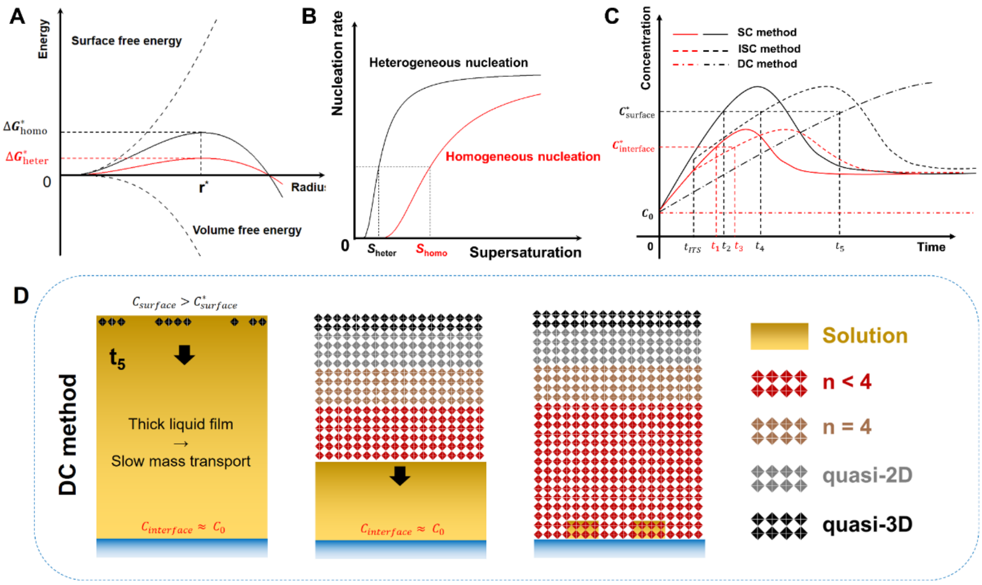

Supplementary Fig. 10 (a) Energy barrier and (b) rate comparison between hetero- and homogeneous nucleation. (c) Evolution of precursor concentration. (d) Crystallization illustrations of DC method.

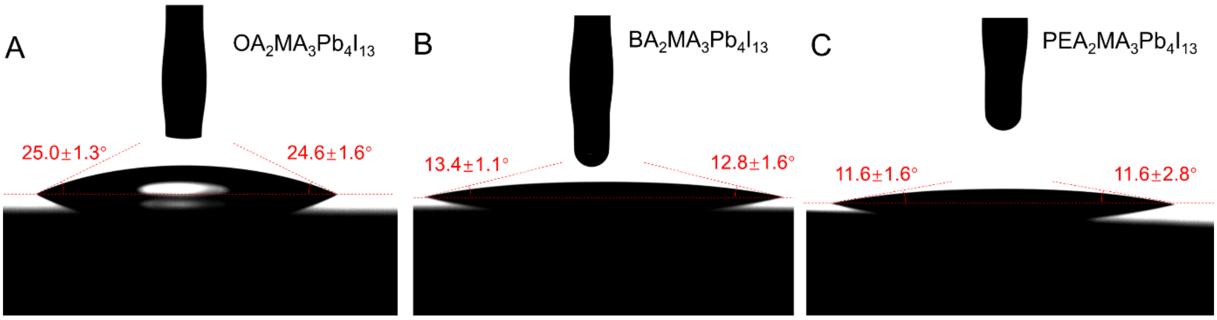

Supplementary Fig. 11 Contact angles between a) OA, b) *n*-BA and c) PEA based 2D PVSK solutions on PEDOT:PSS coated substrate averaged from 3 measurements with standard deviations.

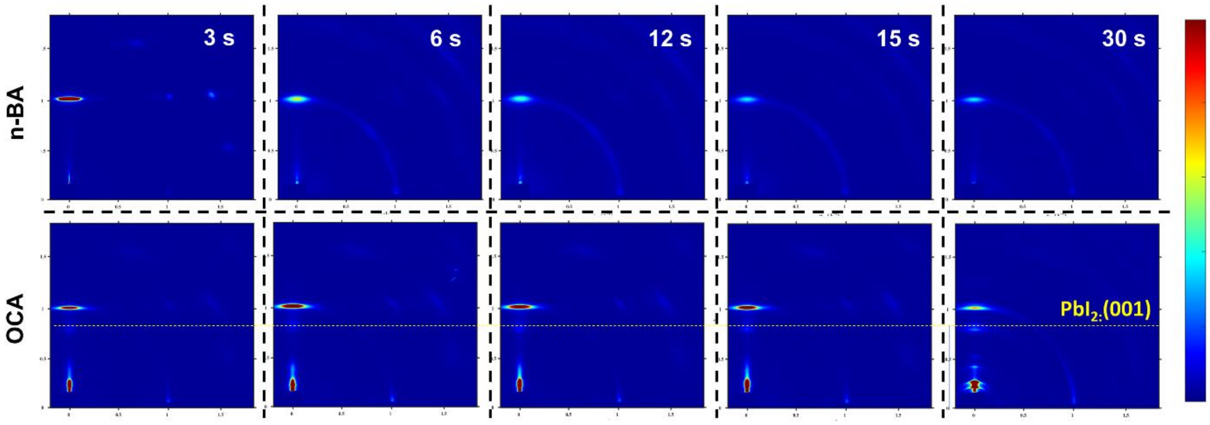

Supplementary Fig. 12 GIWAXS patterns of *n*-BA and OA based 2D PVSKs.

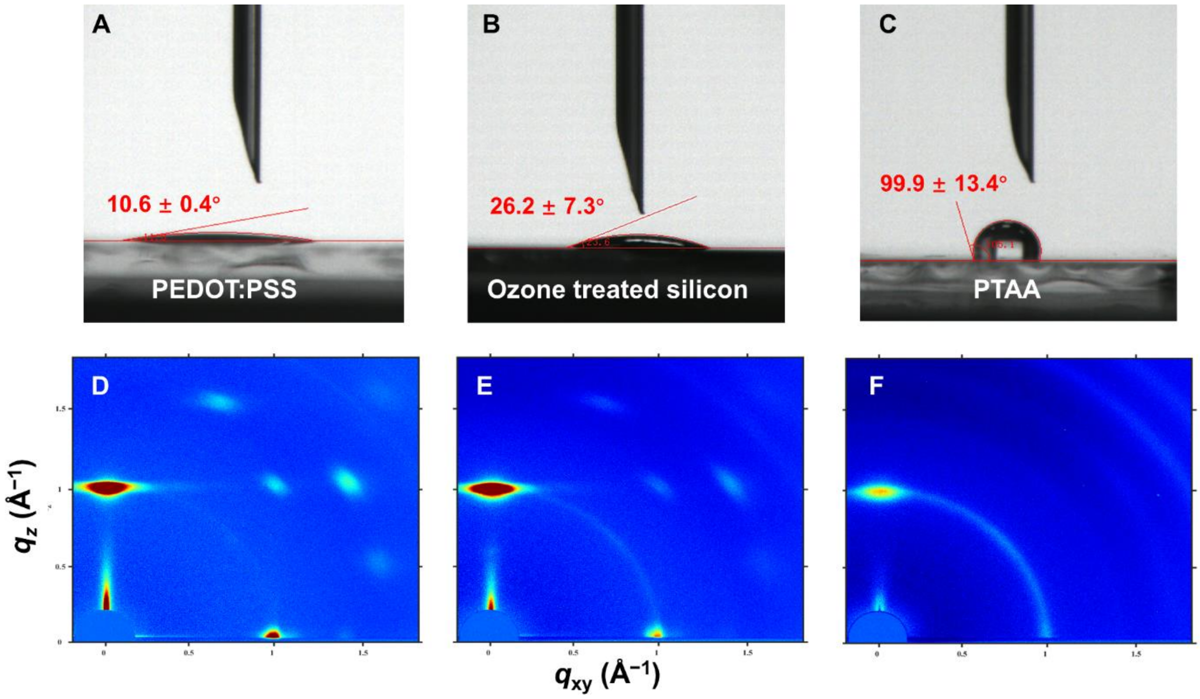

Supplementary Fig. 13 The contact angle measurements (averaged from 3 measurements with standard deviations) and corresponding GIWAXS patterns of $PEA_2MA_3Pb_4I_{13}$ based 2D PVSKs fabricated with ISC-8s method on (a, d) PEDOT:PSS, (b, e) ozone treated silicon and (c, f) PTAA substrates, respectively.

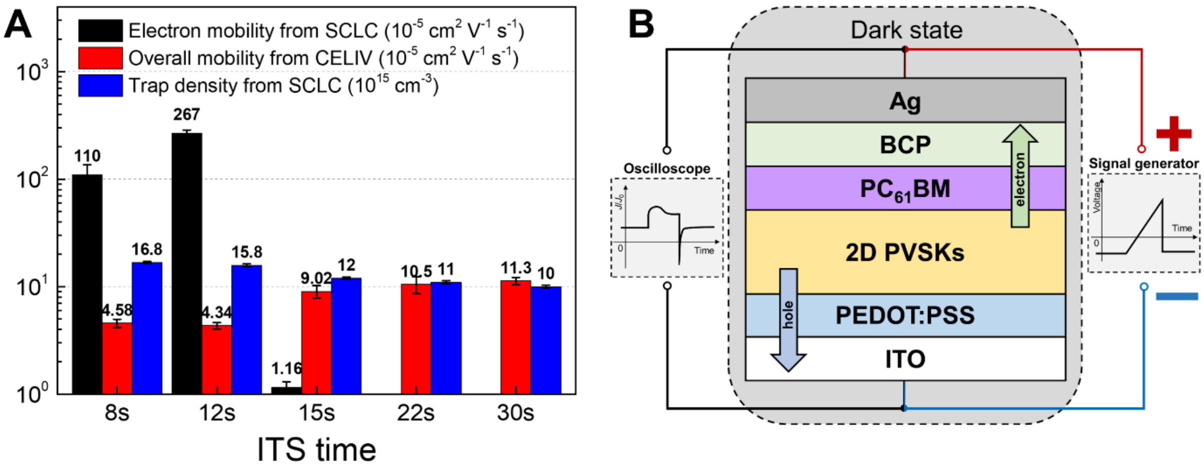

Supplementary Fig. 14 A Statistic results of the mobility and trap density in $PEA_2MA_3Pb_4I_{13}$ thin films (average from 3 devices with standard deviations) via the SCLC and MIM-CELIV methods. B Schematic of MIM-CELIV measurement.

Supplementary Table 2 Fitting parameters of SCLC method.

ISC time	Fitting parameters	Child region	TFL region	Ohmic region
**8s**	Intercept	-14.891 ± 0.110	-47.671 ± 0.065	-13.488 ± 0.604
	Slope	2.004 ± 0.024	9.462 ± 0.16	1.018 ± 0.154

12s	Intercept	-14.589 ± 0.071	-54.329 ± 0.142	-13.569 ± 0.645
	Slope	2.001 ± 0.015	10.901 ± 0.033	1.006 ± 0.161
15s	Intercept	-16.909 ± 0.138	-50.853 ± 0.380	-13.883 ± 0.811
	Slope	2.001 ± 0.029	9.422 ± 0.085	1.000 ± 0.188
22s	Intercept	-16.850 ± 0.208	-55.739 ± 0.313	-13.977 ± 0.939
	Slope	1.998 ± 0.045	10.534 ± 0.070	1.037 ± 0.217
30s	Intercept	-16.848 ± 0.233	-60.947 ± 0.375	-14.111 ± 0.850
	Slope	2.005 ± 0.049	11.540 ± 0.083	1.040 ± 0.194

Note: a) The intercepts and slopes of different region were linearly fitted with standard errors provided.

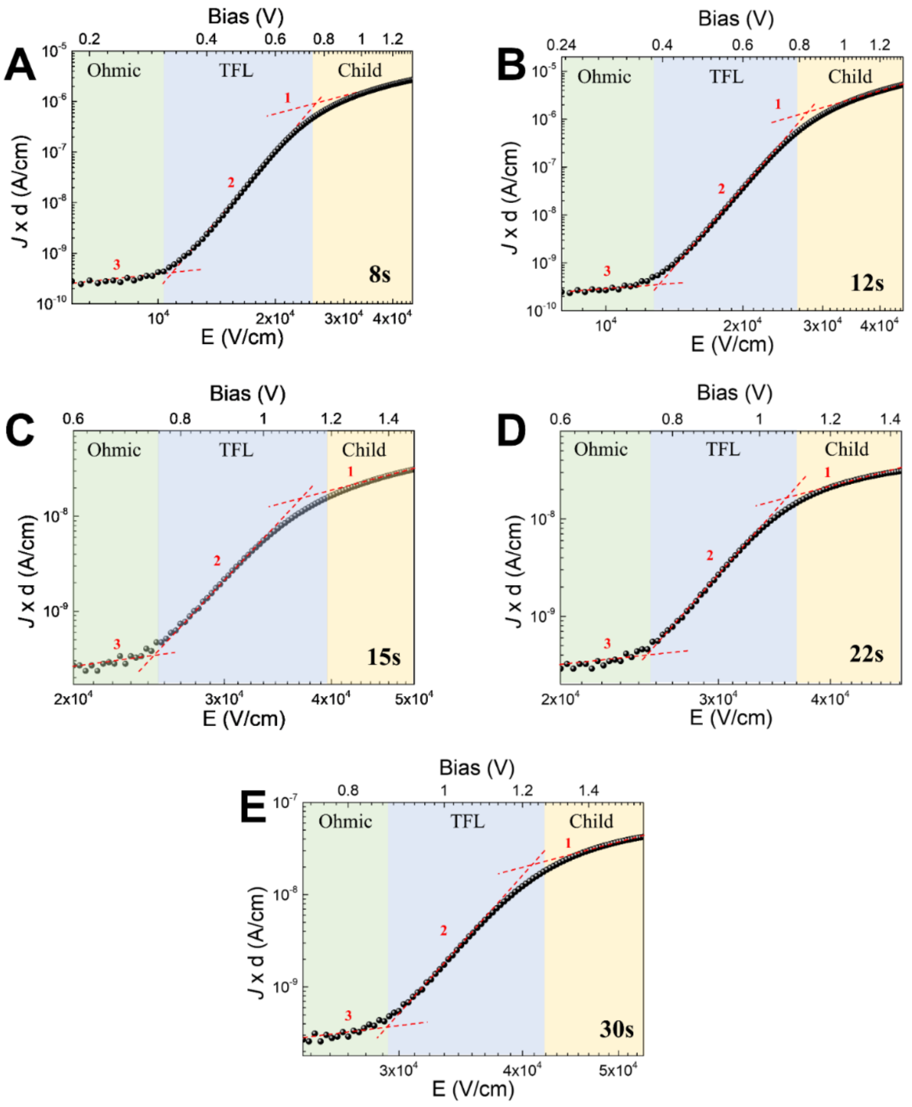

Supplementary Fig. 15 Current vs. electric field characteristics of electron-only devices via SCLC method.

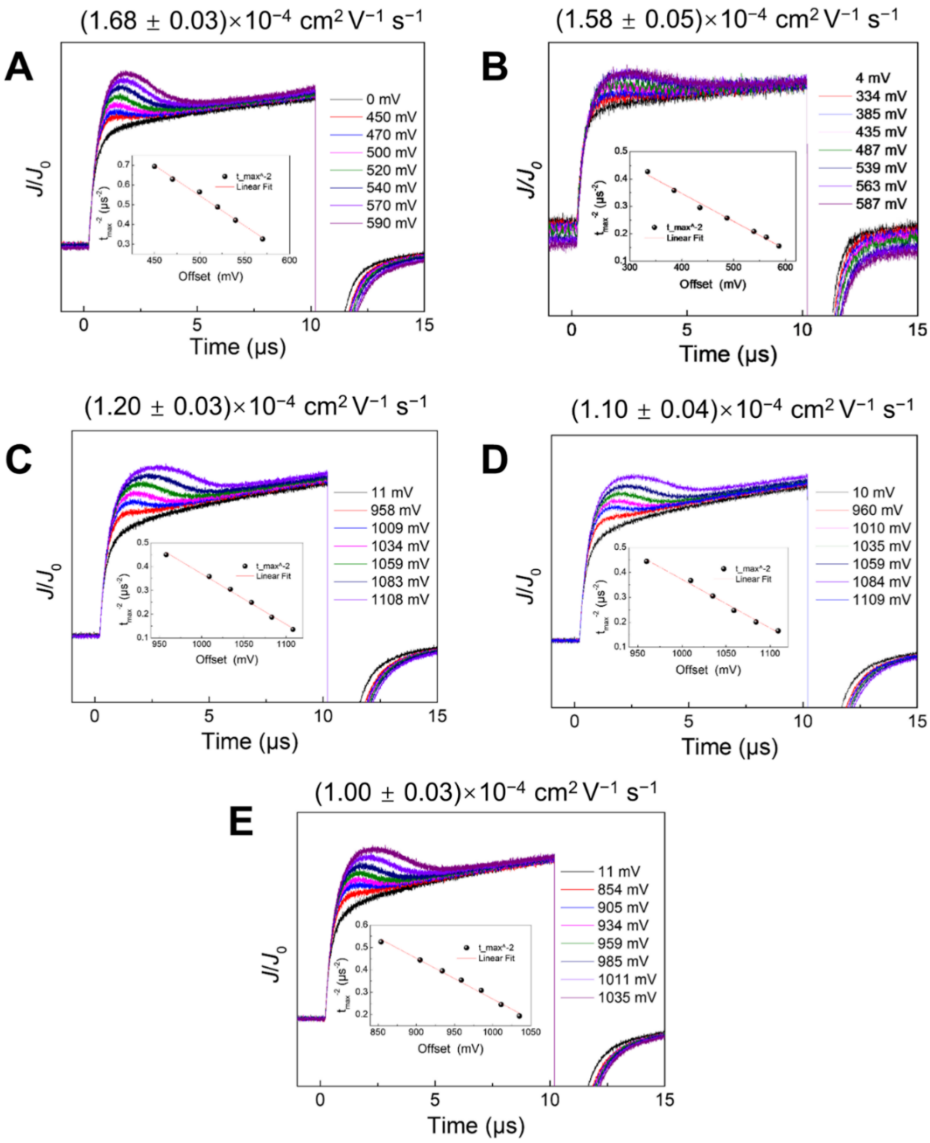

Supplementary Fig. 16 Carrier mobility measurements via CELIV method.

Supplementary Table 3 Efficiency comparison between PEA-based solar cells with *n*-values of 4 and 5.

n-value	Treatment			PCE (%)	Ref.
	Extra solvent	Additive	Preparation method		
5	γ-GBL & TL	N/A	SC	7.8	[1]
	N/A	RbI	SC	14.6	[2]
	DMSO	N/A	SC	9.7	[3]
	N/A	NH_4SCN & NH_4Cl	SC	13.2	[4]
4	DMSO	NH_4Cl	SC	13.4	[5]
	DMSO	MACl	SC	12.1	[6]
	DMSO	N/A	SC	12.2	[7]
	N/A	H_2O & NH_4Cl	VR	17.0	[8]
	N/A	N/A	ISC	11.2	This work
	N/A	NH_4Cl	ISC	14.0	This work

Note: 1) Unless otherwise specified, DMF is used as basic solvent for prepartion precursor solution. 2) Abbreviation: TL = toluene, SC = spin-coating method, VR = vertical rotation method, ISC = intermittent spin-coating method. 3) Structural modification of PEA cation by halogenation such as –F and –Cl is excluded for reasonable comparison.

Supplementary Table 4 Photovoltaic parameters of *n*-$BA_2MA_3Pb_4I_{13}$, $OA_2MA_3Pb_3I_{13}$ and F-$PEA_2MA_3Pb_3I_{13}$ based devices with the ISC-8s and SC methods, respectively

Sample		V_{OC} (V)	J_{SC} (mA/cm^2)	FF	PCE (%)
n-BA ISC-8s	RS	1.07 ± 0.07	12.8 ± 0.8	0.53 ± 0.02	7.38 ± 0.30
	FS	1.02 ± 0.01	13.7 ± 1.0	0.54 ± 0.02	7.17 ± 0.40
n-BA SC	RS	1.05 ± 0.01	5.2 ± 0.5	0.55 ± 0.06	3.00 ± 0.06
	FS	1.03 ± 0.01	5.4 ± 0.5	0.52 ± 0.06	2.91 ± 0.03
F-PEA ISC-8s	RS	0.94 ± 0.14	16.4 ± 1.2	0.51 ± 0.02	7.76 ± 0.30
	FS	0.99 ± 0.04	16.5 ± 1.1	0.49 ± 0.0	7.96 ± 0.08
F-PEA SC	RS	0.93 ± 0.16	5.4 ± 0.1	0.39 ± 0.13	1.91 ± 0.51
	FS	0.89 ± 0.24	5.9 ± 0.1	0.43 ± 0.17	2.26 ± 1.00

Note: a) FS: forward scan, RS: reverse scan. b). The error is provided from 3 independent devices with standard deviations from the average.

References

[1] Ni, C. et al. Thiophene cation intercalation to improve band-edge integrity in reduced-dimensional perovskites. *Angew. Chem. Int. Ed.* **59**, 13977–13983 (2020).

[2] Cui, S. et al. Rubidium ions enhanced crystallinity for Ruddlesden–Popper perovskites. *Adv. Sci.* **7**, 2002445 (2020).

[3] Zhang, F. et al. Enhanced charge transport in 2d perovskites via fluorination of organic cation. *J. Am. Chem. Soc.* **141**, 5972−5979 (2019).

[4] Fu, W. et al. Tailoring the functionality of organic spacer cations for efficient and stable quasi-2D perovskite solar cells. *Adv. Funct. Mater.* **29**, 1900221 (2019).

[5] Yu, S. et al. Enabling room-temperature processed highly efficient and stable 2D Ruddlesden–Popper perovskite solar cells with eliminated hysteresis by synergistic exploitation of additives and solvents. *J. Mater. Chem. A* **7**, 2015–2021 (2019).

[6] Qing, J. et al. Aligned and graded type-II Ruddlesden–Popper Perovskite films for efficient solar cells. *Adv. Energy Mater.* **8**, 1800185 (2018).

[7] Wang, Z. et al. Spacer cation tuning enables vertically oriented and graded quasi-2D perovskites for efficient solar cells. *Adv. Funct. Mater.* **31**, 2008404 (2021).

[8] Yang, Y. et al. Universal approach toward high-efficiency two-dimensional perovskite solar cells via a vertical-rotation process. *Energy Environ. Sci.* **13**, 3093–3101 (2020).
