# Supplementary material for: Implementing an intermittent spin-coating strategy to enable bottom-up crystallization in layered halide perovskites
Source: Nat Commun. 2021 Nov 15;12:6603. doi: 10.1038/s41467-021-26753-3 (PMC8593150; doi:10.1038/s41467-021-26753-3)
Supplement: Supplementary file 2 — Description of Additional Supplementary Files [file 41467_2021_26753_MOESM2_ESM.pdf]

Description of Additional Supplementary Files

File Name: Supplementary Movie 1
Description: In-situ GIWAX 2D pattern evolution of surface area in ISC-8s sample recorded with 2 frames per second.

File Name: Supplementary Movie 2
Description: In-situ GIWAX 2D pattern evolution of bulk area in ISC-8s sample recorded with 2 frames per second.

File Name: Supplementary Movie 3
Description: In-situ GIWAX 2D pattern evolution of surface area in SC sample recorded with 2 frames per second.

File Name: Supplementary Movie 4
Description: In-situ GIWAX 2D pattern evolution of bulk area in SC sample recorded with 2 frames per second.

File Name: Supplementary Movie 5
Description: Preparation process of ISC-8s sample.

File Name: Supplementary Movie 6
Description: Preparation process of SC sample.
